# All-Atom Molecular Dynamics Simulations of Electrotunability in Ionic Liquid Lubrication: Mechanism and Anion Specificity

**DOI:** 10.1021/acs.jpcc.6c03564

**Published:** 2026-08-13

**Authors:** Md Ashiqur Rahman, Wei Song, Ejaz Ahmed, Huajie Zhang, Akam Aboubakri, Rosa M. Espinosa-Marzal, Rui Qiao

**Affiliations:** † Department of Mechanical Engineering, Virginia Tech, Blacksburg, Virginia 24061, United States; ‡ Department of Civil and Environmental Engineering, 14589 University of Illinois Urbana–Champaign, Urbana, Illinois 61801, United States; § Department of Materials Science and Engineering, University of Illinois Urbana–Champaign, Urbana, Illinois 61801, United States

## Abstract

We report an all-atom molecular dynamics study of the electrotunable friction of ionic liquids in systems featuring a single ion layer confined between a hemispherical model silica tip and a flat graphene substrate. The ion specificity of friction is probed through two ionic liquids with the same cation, [EMIM]^+^, but different sulfonyl-based anions, [TFSI]^−^ and [TfO]^−^. We show that the friction force vs surface charge density is V-shaped, with minimum friction occurring at −0.12 C/m^2^. In the curves’ right branch, friction for [EMIM]­[TfO] is considerably smaller than for [EMIM]­[TFSI]. In the left branch, the friction for the two ionic liquids shows only a minor difference. Analysis reveals that the instantaneous friction force exhibits steady rises and sudden drops due to the stick–slip motion of the tip-bound ion layer over the graphene substrate. Such a stick–slip response arises from ions becoming temporarily trapped in, and subsequently escaping from, the energy valleys associated with nearby graphene atoms. The number of trapped ion atoms is identified as the decisive factor governing the friction force. Quantifying and then tracing them to the composition and orientation of ions confined under the tip apex enables a mechanistic understanding of the observed electrotunability of friction and its ion specificity.

## Graphical abstract

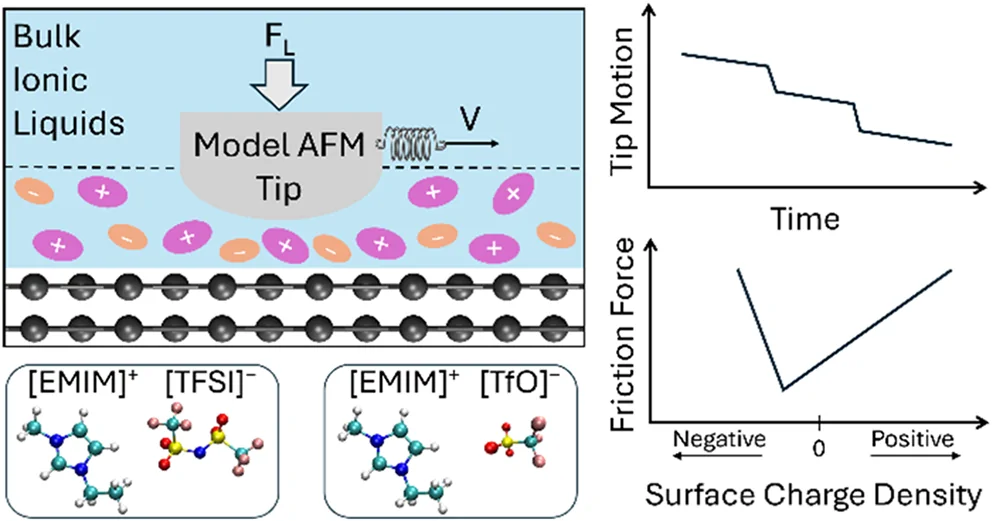

## 1 Introduction

Ionic liquids were first proposed as lubricants in the early 2000s in part due to their unique thermophysical properties, including ultralow volatility, high thermal resistance, nonflammability, low melting temperature, and broad compositional flexibility.^1–3^ Because of their strong surface adsorption, ionic liquids resist squeeze-out, allowing significantly higher loads to be sustained than those with conventional lubricants.^4–6^ Moreover, it has been discovered that the application of an electric field can modulate the frictional response.^7–9^ Experimental investigations on the electrotunability of ionic liquid-based lubrication demonstrate that the applied electrical field induces a structural reorganization of ions confined in the nanogap formed between solid substrates, which directly controls friction behavior.^7,10–13^

The structure of ionic liquids in nanoconfinement and their modulation of friction behavior has been extensively studied using Molecular Dynamics (MD) simulations, primarily through coarse-grain models, to elucidate the mechanisms governing friction and its electrotunability. Ionic liquids under confinement develop distinct layered density profiles, with the highest concentration of ions typically observed in the immediate vicinity of the confining substrates.^14–16^ The structural organization of these confined films is dictated by a complex interplay between polarity and magnitude of surface charge, ion size and shape, and ion configuration relative to other ions and the substrate.^17–19^ For instance, at neutral or low negative surface charges, the aromatic rings of imidazolium-based cations tend to orient parallel to the substrate, but progressively tilt toward a more vertical configuration as the negative surface charge increases.^20–22^ Variation of ion size can introduce complex effects on tribological performance.^23^ Shorter chain imidazolium cations slide more easily than longer ones and better resist being squeezed out, giving much lower friction.^20^ In contrast, larger anions (e.g., [TFSI]^−^) adsorbed on graphene substrates can exist in a variety of configurations relative to the substrate, which leads to a broader density peak near the graphene and increased instability and higher friction under shear.^21,24,25^ Conversely, the smaller anion builds alternating charged layers which slide past each other under shear, leading to reduced instability and, consequently, lower friction.^20,25^

Previous experimental and computational works have advanced our understanding of the friction mechanism and the electrotunability feature of ionic liquids as lubricants. However, most simulation efforts have considered nanoconfined systems mimicking the configuration in Surface Force Apparatus (SFA), whereas many experimental investigations of ionic liquid electrotunability employed Atomic Force Microscopy (AFM).^18^ In this context, Pivnic and colleagues^18^ examined electrotunable friction of a model AFM tip above graphene substrates mediated by [BMIM]­[PF_6_] through MD simulations. They adopted coarse-grained models to describe the ionic liquids but full atomistic models for the graphene and AFM tip. They observed that, when the strength of the van der Waals interactions between ionic liquids and the graphene substrate is taken from the work of Cole and Klein,^26,27^ the friction force vs graphene surface charge density curve is V-shaped with minor asymmetry around the minimum at zero surface charge density, for the surface charge densities considered (0, ±0.16, and ±0.32 C/m^2^). The observed electrotunability was traced to the modulation of the composition and orientation of ions in the nanogap beneath the model AFM tip.

The work by Pivnic and colleagues^18^ provided significant insights into the electrotunability of ionic liquid-mediated friction and also left some open questions. For example, to what extent coarse-grained models of ionic liquids capture the friction modulated by single ion layers, where molecular details may affect the energy landscape experienced by ions? This question was partially addressed in our recent simulation work, where we examined the friction between a model AFM tip and graphene substrate mediated by [EMIM]­[TFSI] through all-atom MD simulations.^28^ Our simulations revealed a V-shaped friction vs surface charge curve similar to that found by Pivnic and colleagues.^18^ However, our previous work primarily focused on the macroscopic frictional response, with limited insight into the underlying mechanisms and key factors governing friction modulation. For example, the molecular structure of ionic liquids in the nanogap above graphene was not thoroughly quantified. The assessment of correlations between the movement of nanoconfined ions, the tip’s translation motion, and the friction force, as well as how these movements and forces are governed by the composition and structure of ions under the model AFM tip, was lacking. Moreover, the ion specificity of the electrotunability of friction was also not probed.

In the current work, the mechanism of electrotunable friction in ionic liquids is investigated using an all-atom MD simulation featuring a hemispherical tip and graphene substrate. Two ionic liquids with sulfonyl-based anions (1-ethyl-3-methylimidazolium bis­(trifluoromethylsulfonyl)­imide, [EMIM]­[TFSI]; 1-ethyl-3-methylimidazolium trifluoromethanesulfonate, [EMIM]­[TfO]) are used to elucidate how different anions affect friction. The remainder of this manuscript is organized as follows. Section 2 describes the simulation system and methods. In Section 3, the structure and friction mechanism of [EMIM]­[TFSI] are explored first for a reference case with a graphene charge density of +0.24 C/m^2^, which is followed by an analysis of the electrotunability of friction for the same ionic liquid. A comparative study of the frictional behavior of [EMIM]­[TFSI] and [EMIM]­[TfO] was then presented. Finally, conclusions are drawn in Section 4.

## 2 Simulation Systems, Models, Protocol, and Methods

### 2.1 Simulation System

This study employs all-atom molecular dynamics simulations to investigate the electrotunability of frictional forces in an ionic liquid lubricated system, focusing on how the substrate charge can control nanoscale friction. The simulation system is similar to our previous work,^28^ and its snapshot is provided in Figure 1, with a portion of the ionic liquid hidden to improve clarity. The simulation models a typical AFM experimental setup consisting of a graphite substrate and a model hemispherical silica tip.^10,12,18^ The graphite substrate is modeled as two AB-stacked graphene layers separated by a distance of 0.335 nm with a lattice parameter of 0.24 nm. Following the practice by Pivnic and colleagues,^18^ a hemispherical FCC crystalline silica tip with a lattice spacing of 0.36 nm and an apex radius of 1.4 nm is placed above the substrate.

**Figure 1:**
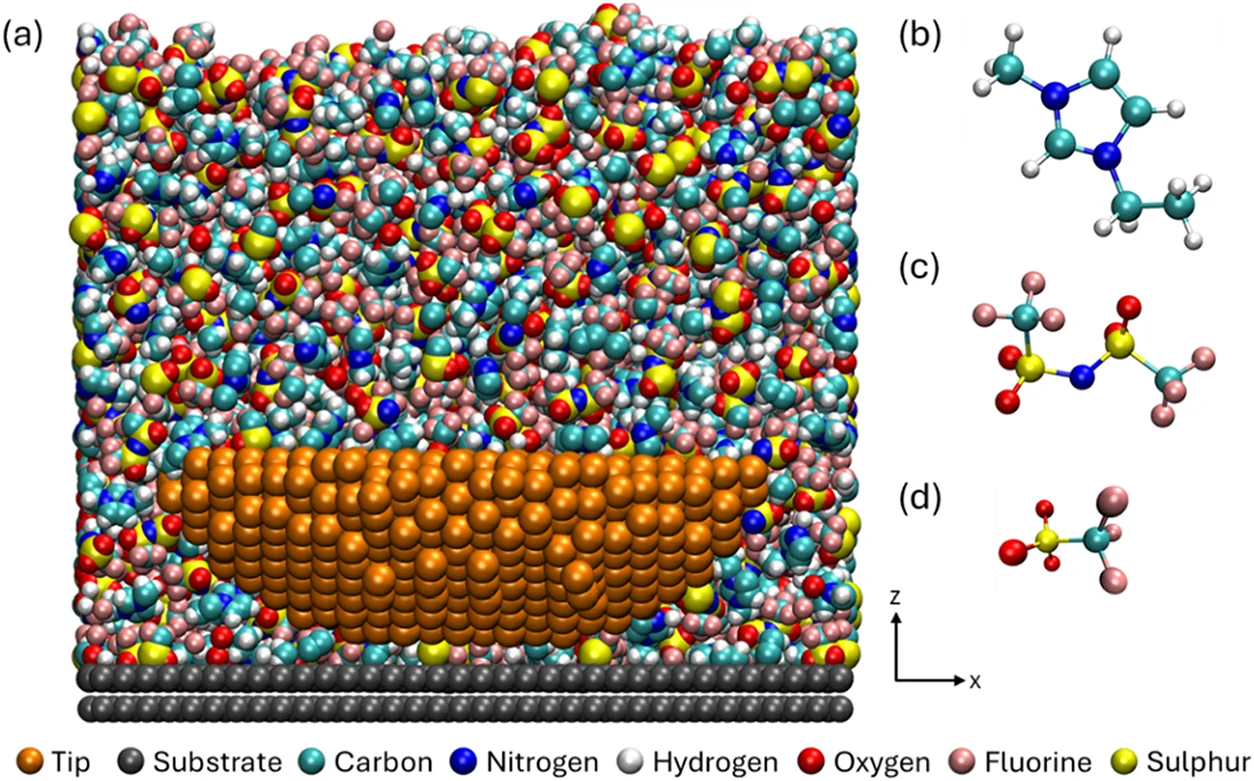
Snapshot of the molecular system used to study the electrotunability of ionic liquid-mediated lubrication. (a) The full system, showing the [EMIM]­[TFSI] ionic liquid as well as the tip and graphene atoms, colored in orange and gray, respectively. (b) [EMIM]^+^ cation. (c) [TFSI]^−^ anion. (d) [TfO]^−^ anion.

To investigate the influence of anion size on tribological properties, we conducted MD simulations of two ionic liquids: [EMIM]­[TFSI] and [EMIM]­[TfO]. These ionic liquids are chosen due to their widespread use in experimental studies and the significant difference in the size and geometry, despite both featuring sulfonyl-based anions.^29–31^ The [TFSI]^−^ ion, with a molecular volume of about 0.221 nm^3^, is bulkier than [TfO]^−^ ion (molecular volume: 0.117 nm^3^). By isolating the size difference between the anions while keeping the cation [EMIM]^+^ consistent, we can specifically analyze how structural variation affects the friction within the system. Ionic liquids are introduced into the simulation box that measures 8.39, 8.12, and 10 nm in the *x*-, *y*-, and *z*-directions. Periodic boundary conditions are applied in the *x-* and *y-*directions.

Multiple simulations are performed to explore the effect of substrate charge density, ranging from −0.24 C/m^2^ to +0.24 C/m^2^, on the system’s tribological behavior. This range encompasses the regime where significant changes in ionic layering, interfacial structure, and friction occur, consistent with previous MD studies, and the general electrotunable friction trends computed within this range align well with those obtained from AFM measurements.^18,28^ To create a desired surface charge density, a uniform partial charge is assigned to all carbon atoms of the uppermost layer of the graphene substrate. The tip is maintained in a neutral state, as Pivnic and colleagues reported that the friction is not sensitive to the charge of the tip.^18^ The ions in the bulk region function as a reservoir for the ions in the nanoscale gap under the tip. The exchange of ions between these two regions is influenced by the surface charge density, temperature, and normal load. When the substrate is charged, the number of ions in the system is adjusted accordingly to ensure overall system neutrality.

### 2.2 Molecular Model

The graphene substrate is prepared using Charmm-GUI server, which use INTERFACE force field.^32,33^ The bottom graphene layer of the substrate is kept frozen for all of the simulations, while the topmost graphene layer is not frozen. This approach represents a mechanically supported substrate while allowing the top layer to capture local flexibility and thermal vibrations. Since the contact region is very small (the tip apex radius is only 1.4 nm), graphene’s flexibility should cause only minute changes in graphene atom position and thus have a negligible effect on friction. The tip is modeled as a face-centered cubic (FCC) hemispherical structure with an effective LJ diameter of 0.3218 nm and a LJ potential with an interaction strength of 50 kJ/mol to maintain the rigidity of the tip.^18^ Ionic liquids are described using the all-atom force fields developed by Acevedo and colleagues,^34,35^ which have been shown to reproduce the structural and dynamic properties of ionic liquids well. LJ parameters of tip-ionic liquid interactions are determined using the Lorentz–Berthelot combination rule. Such a choice results in a rather strong adhesion of ionic liquids to the tip. This choice is reasonable because our tip is a simplified structure composed solely of LJ atoms, whereas experimental studies typically use amorphous silica tips whose surface atoms generally have large partial charges and therefore strongly interact with ionic liquids. Since the latter are not explicitly captured in our simplified LJ tip model, tip-ionic liquid interactions need to be strong to compensate for missing tip-ionic liquid electrostatic interactions and to create a realistic representation of ion adsorption at the tip surface.

### 2.3 Simulation Protocol

Initially, the tip is placed at a distance of 1.7 nm above the graphene substrate’s topmost layer. The system is then equilibrated for 10 ns in the NVT ensemble to achieve a stable starting configuration. Subsequently, ionic liquids are introduced, and the entire system is equilibrated for an additional 10 ns. This two-step equilibration process is performed to ensure that both solid–solid and solid–liquid interfaces are properly relaxed and that the entire system reaches a stable, low-energy state before any forces are applied.

After the equilibration described above, a constant downward load is applied to the tip. This step is crucial for confining ionic liquids between the tip and substrate. Here, a normal load of 28.8 nN was applied for 60 ns to press the tip downward. During this process, the tip is held rigid in the *x*- and y-directions to prevent any tilting. As the tip moves downward, excess ionic liquid layers are displaced, resulting in a stable one-layer film between the tip and the substrate. Such a single-ion-layer configuration is similar to that which has been studied in the literature to understand the electrotunability of ionic liquids.^8,16,36^

Once the single-layer configuration is achieved, the tip is subjected to a lateral sliding. This is simulated by pulling the tip in the *x*-direction with a virtual spring of 5 N/m stiffness. The tip was pulled at a constant sliding velocity of 1 nm/ns for 80 ns. The vertical load remains active throughout this stage, and the tip’s movement is restricted in the *y* direction. This stage of the simulation is specifically designed to measure the frictional force during the lateral sliding.

### 2.4 Simulation Methods

All simulations are performed using the GROMACS 2024.3 code in the canonical (NVT) ensemble with a time step of 2 fs.^37^ For packing the substrate, tip, and ionic liquids into the system, the PACKMOL code is used.^38^ The temperature of solid components (tip and the uppermost graphene layer) is maintained at 450 K using the velocity rescale thermostat^39^ with a coupling time constant of 1 ps. Although the ionic liquid’s temperature was not controlled directly, it was monitored and consistently found to be in thermal equilibrium with the solid components, ensuring that the entire system is at a stable and consistent temperature. The elevated temperature ensures sufficient ionic mobility by reducing the viscosity and accelerating structural relaxation while preserving the essential interfacial structure and dynamics of ionic liquids.

Nonbonded interactions are handled using the Verlet cutoff scheme, with a cutoff distance of 1 nm. To ensure accurate representation of Coulombic forces, electrostatic interactions are calculated using the Particle Mesh Ewald (PME) method. For this, a real-space cutoff of 1 nm, an FFT spacing of 0.12 nm, and a relative accuracy of 10^–5^ are used.

## 3 Results and Discussion

We begin by investigating one representative case (substrate surface charge density: +0.24 C/m^2^; [EMIM]­[TFSI] as lubricant) to understand the essential features and mechanisms of friction on an electrified graphene substrate. We next examine how friction is modulated by the surface charge density of graphene, again with [EMIM]­[TFSI] as the lubricant. Finally, a comparative analysis of the friction in the cases of [EMIM]­[TFSI] and [EMIM]­[TfO] as lubricants is presented.

### 3.1 Friction Modulated by [EMIM]­[TFSI] at σ = +0.24 C/m^2^

In the system considered here, friction is governed by ion–substrate, ion–ion, and ion–tip interactions in the region below the tip apex, which is in turn controlled by the composition, distribution, packing, and orientation of ions in this region. We thus examined these composition and structural details of ions below the tip apex.

Figure 2a shows that the [TFSI]^−^ ions dominate this region, although a small number of cations do exist. Such an ion composition is consistent with the positive surface charge density of the graphene substrate and strong cation–anion correlations in ionic liquids, as have been reported in previous studies of ionic liquids confined in nanopores.^40,41^ Note that although two peaks are observed for [TFSI]^−^ ions, the distance between the peaks, ≈2 Å, is too small to support two distinct layers. Instead, these peaks are attributed to different orientations, a common occurrence for the bulky [TFSI]^−^ anion.^21^ The [EMIM]^+^ ions, with their density peak located between the two [TFSI]^−^ peaks, are dispersed between the [TFSI]^−^ ions. These [EMIM]^+^ ions mostly lie flat on the substrate, as observed in the probability density of the orientation of cations’ imidazolium rings relative to the normal vector of the graphene substrate (Figure 2b).

**Figure 2:**
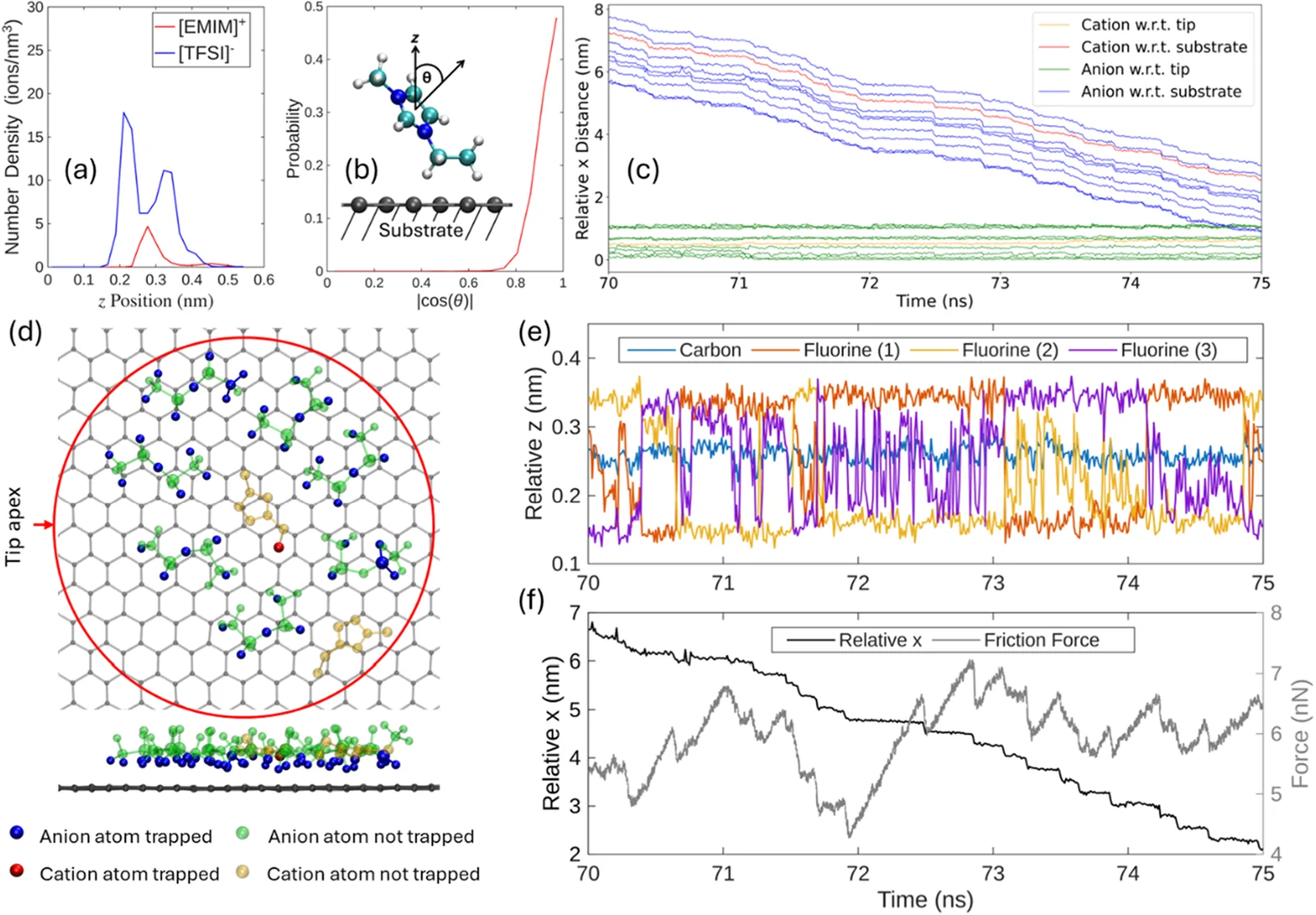
(a) Number density of cation and anion. Cation is located by its ring center, and anion is located by its nitrogen atom. (b) Probability density of cation ring orientation, where θ is the angle between the normal of the cation ring and the normal of the substrate (*z-*axis). (c) Relative *x*-distance of ions beneath the tip apex with respect to both the tip center and a fixed reference substrate atom. (d) Spatial configuration of ions beneath the tip apex in conjunction with their depth (hydrogen atoms and ions that are partially located beneath the tip apex are not shown). The top panel shows the top view, and the bottom panel shows the side view. (e) Relative *z*-position of atoms within a CF_3_ group, measured from the upper substrate. (f) Relative *x*-position of the tip with respect to a fixed atom in the substrate and the corresponding instantaneous friction force with respect to time.

Having clarified the composition and orientation of ions under the tip, we now explore the movement of these ions, which directly influence the friction force. To this end, the ions located beneath the tip apex, which is pulled in the *x*-direction at a constant velocity of 1 nm/ns at *t* = 70 ns, are selected. We then compute their displacement in the *x*-direction relative to the tip center and a fixed reference point on the substrate for 5 ns. Figure 2c shows that the relative distance between the ions and the tip remains almost the same over time with only minor fluctuations, indicating a no-slip behavior of ions over the tip apex. In contrast, the relative distance between the ions and the fixed reference atom on the substrate decreases with time. This shows that the slip plane is located at the ion–substrate interface. This observation is similar to those reported in some previous studies of the lubrication of graphene substrates by ionic liquids^12,18^ but differs from the slip plane at the ion–ion or ion–tip interface reported in some other studies.^22,42^ This suggests that the location of the slip plane is not universal. Instead, it likely depends on factors such as the number of ionic liquid layers in the gap, the magnitude of the applied normal load, and the structure and composition of the tip, which governs ion–tip interactions.

A more careful study of Figure 2c reveals that the *x*-displacement curves of ions under the tip relative to the substrate exhibit a staircase-like profile. Such a profile is indicative of a stick–slip motion of the ions under the tip, consistent with some prior reports.^13,43^ Concurrently, the friction force exhibits a sawtooth profile. A notable observation for the stick–slip motion in our system is its synchronous nature, i.e., the ions under the tip stick to it and slip over the substrate together (this is seen clearly by tracking the relative *x*-displacement of the constituent atoms of cations and anions under the tip, see Figure S1). Such a kind of motion is caused by the temporary trapping of ions in the energy valley located at the interstitial space between graphene atoms. Specifically, an ionic liquid atom entering an energy valley remains trapped until sufficient energy is accumulated to overcome the energetic barrier of the trap, corresponding to the stick phase when friction increases. Upon accumulating the requisite energy, the atom undergoes a translational jump rapidly to get out of the trap, a phenomenon described as slip when the friction force drops.

The trapping of ionic liquid atoms in energy valleys can be visualized using snapshots and the time evolution of their *z*-positions (Figure 2d,e). For example, we select a CF_3_ group of a [TFSI]^−^ ion under the tip apex and track the *z*-positions of its four atoms (see Figure 2e for a compact representation; the individual atomic *z*-positions are shown in Figure S2 for clarity). Figure 2e shows that the *z*-movement of the carbon atom is insignificant. However, the *z*-position of the three fluorine atoms exhibits periodic jumps, with a nanosecond wait period in between. When an atom’s *z*-position falls below a certain threshold and then remains there for an extended time, it is reasonable to take the atom as “trapped”. By examining the *z*-position of ion atoms beneath the tip and correlating it with trajectory visualization (for details, see the Supporting Information), we find that this threshold is between 0.22 and 0.26 nm, depending on the atom type. Using this criterion and the *z*-position evolution in Figure 2e, we find that several fluorine atoms of the same [TFSI]^−^ ion can be in the “trapped” state simultaneously. When one of the trapped atoms jumps out of a trap (i.e., its *z*-position increases rapidly), other atoms of this group may show significant *z*-movement (e.g., jumps into a trap and its z-position decreases rapidly) or little *z*-movement. The latter indicates that atoms of an ion can get into or out of energy valleys through an ion’s rotational motion.

Figure 2d shows representative snapshots of the ions under the tip. Many anion atoms are trapped in the energy valley between graphene atoms, whereas only one cation atom (colored in red) is found to be trapped in the energy valley and at a position shallower than the trapped anion atoms. The latter is consistent with the coplanar configuration of cations on graphene, which prevents atoms of the rather planar [EMIM]^+^ ions from penetrating deep into the interstitial spaces between graphene’s carbon atoms, where energy valleys exist.^18^

Finally, the movement of the tip and the corresponding instantaneous friction force are studied. Figure 2f shows that a close correlation between these two phenomena exists, i.e., the stick–slip motion of the tip is generally accompanied by sudden changes in the friction force, consistent with observations reported in previous studies.^10,44,45^ We also performed simulations with a sliding velocity of 0.1 nm/ns and confirmed that this correlation persists at a lower sliding velocity (see Figure S3). When ion atoms below the tip are trapped in energy valleys between graphene atoms, the movement of the tip is obstructed. This obstruction is attributed to the ions adsorbed to the tip (cf. the no slip between ions and tip revealed in Figure 2c). During this “stick” period, the instantaneous friction force continues to increase (or climb). When sufficient energy is accumulated, the trapped ions are dislodged from the energy valleys (Figure 2e), resulting in a sudden slip of the tip. As this slip occurs, a jump (reduction) in the friction force is noticed. The strong alignment between the stick–slip motion of ions/tip with the jumps of friction force suggests that the trapping of ion atoms in energy valleys and their escape are crucial energy dissipation mechanisms and thus a key source of friction loss. This, along with the observation that most atoms trapped under the tip apex are anion atoms, supports the dominant role of anions in controlling the friction in the systems considered here.

### 3.2 Electrotunability of Friction in [EMIM]­[TFSI]

The frictional behavior of [EMIM]­[TFSI] under varying surface charge densities is explored, and the result is presented in Figure 3a (friction of [EMIM]­[TfO] is also shown and will be discussed later). Although this frictional behavior was reported in our previous work,^28^ the underlying mechanisms and key factors governing its modulation were not addressed and constitute the primary focus of the present study. The maximum friction, 5.98 nN, occurs at a surface charge density of +0.24 C/m^2^, the case discussed in Section 3.1. As the graphene surface charge density, σ, decreases, the friction decreases, and it continues until σ reaches −0.12 C/m^2^, where a minimum friction of 2.27 nN is recorded. As σ becomes more negative, the friction starts to increase and reaches a high friction of 5.61 nN at σ = −0.24 C/m^2^, which is comparable to the maximum friction observed at +0.24 C/m^2^. The V-shaped response of friction to the electrification of graphene is similar to that observed in coarse-grained MD simulations of friction at a graphene substrate immersed in [BMIM]­[PF_6_].^18,46^ However, in our system, the minimum friction does not occur at the neutral substrate; instead, it is observed at σ = −0.12 C/m^2^. Below, we clarify the variation of friction for the positive and negative branches of the substrate surface charge density.

**Figure 3:**
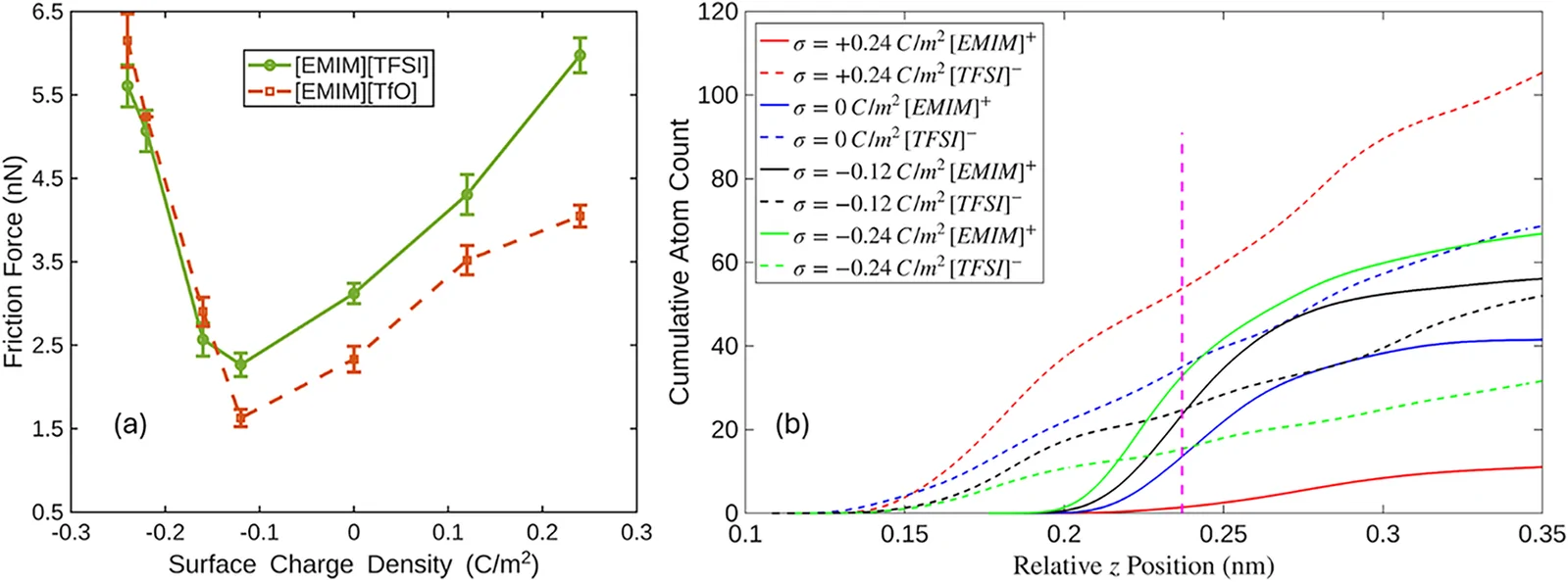
(a) Variation of friction force with net surface charge density of graphene for ionic liquids [EMIM]­[TFSI] and [EMIM]­[TfO]. (b) The cumulative count of the number of cation and anion atoms beneath the tip as a function of the distance from the graphene surface for different surface charge densities and ionic liquids. The vertical dashed line indicates the approximate location of the trapping boundary (atoms on the left of this line are considered trapped in the energy valleys between graphene atoms).

#### 3.2.1 Friction at σ ≥ 0

It is already discussed that when the surface charge is highly positive (σ = +0.24 C/m^2^), few cations exist under the tip and their atoms cannot penetrate deep into the energy valleys on the graphene substrate because of their coplanar orientation, and the friction is dominated by anion. As graphene’s surface charge density is reduced from +0.24 C/m^2^ to +0.12 C/m^2^, and subsequently to 0, the number of anions decreases gradually (see Figures 4a,b and 2a). Meanwhile, the number of cations under the tip increases. However, cations’ imidazolium rings remain mostly parallel to the graphene substrate (see Figure 4c), making their deep trapping by the energy valleys difficult. This is corroborated by Figure 3b, which shows the cumulative count of cation and anion atoms beneath the tip as a function of their *z*-position relative to the graphene substrate. We observe that, at σ = 0 C/m^2^, only a small number of cation atoms are trapped in the energy valleys near the graphene (i.e., their *z*-position is less than about 0.24 nm). Overall, as the surface charge density decreases from +0.24 C/m^2^ to 0, the contribution of cations to friction remains modest, while the contribution from anions drops greatly. Therefore, the friction force is observed to diminish gradually as the surface charge is reduced from +0.24 C/m^2^ toward neutral.

**Figure 4:**
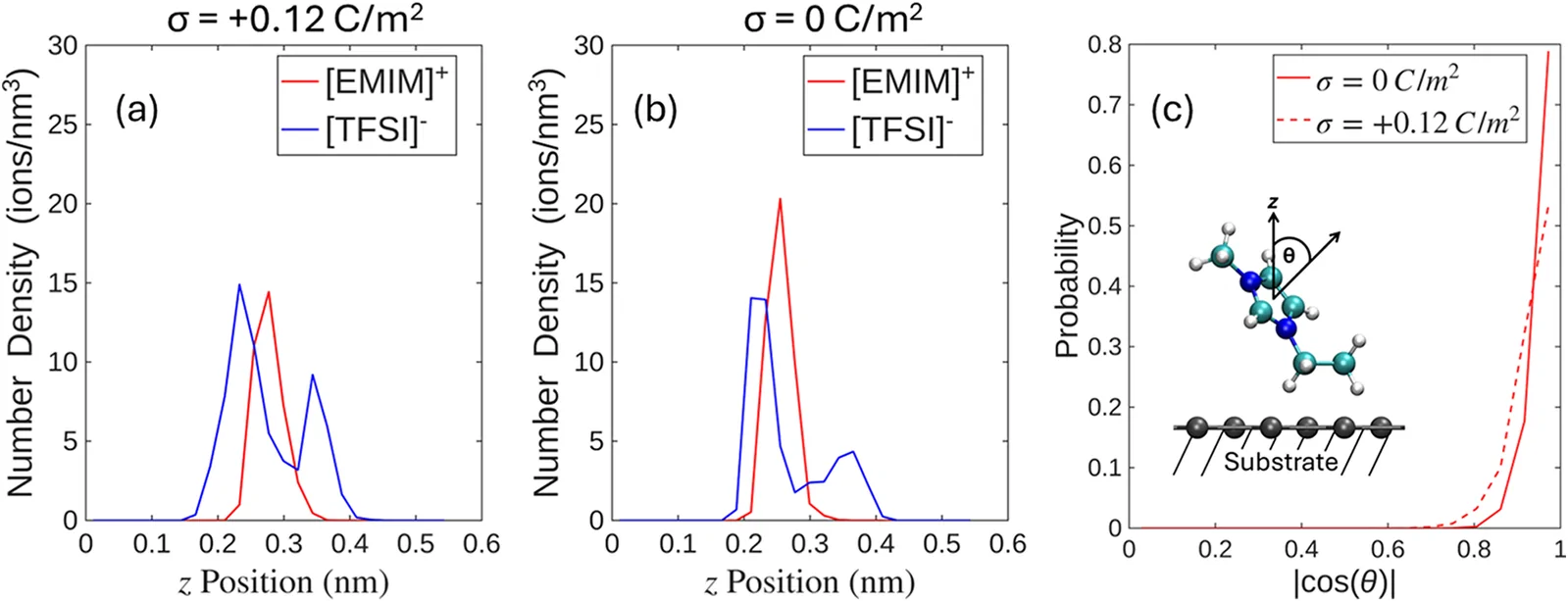
Number density profile of cations and anions across the nanogap formed by the graphene substrate and the tip apex for graphene surface charge densities of σ = +0.12 C/m^2^ (a) and σ = 0 (b). (c) Comparison of the orientation of the imidazolium rings of cations trapped beneath the tip when the substrate charge is +0.12 and 0 C/m^2^.

#### 3.2.2 Friction for σ ≤ 0

As the surface charge density of the graphene substrate decreases from 0 to −0.12 C/m^2^, the friction decreases further. To understand this, we first examined the position of the slip plane at σ = −0.12 C/m^2^ (see Figure S5) and found it to remain the same as before, i.e., at the ion–substrate interface. Next, we examined the composition and structure of ions in the gap between the tip apex and substrate. Figure 5a shows that there is still a single layer of ions under the tip at σ = −0.12 C/m^2^, and the cation (anion) density under the tip apex becomes higher (lower) than when the graphene is neutral. Furthermore, the cation rings still adopt a nearly coplanar orientation relative to the graphene (see Figure 5c). Consequently, although the number of cations under the tip does increase notably, the number of cation atoms trapped in the energy valleys near graphene atoms increases rather modestly (see Figure 3b). Meanwhile, the number of anion atoms trapped in the energy valley decreases significantly compared to the neutral substrate (see Figure 3b). Because the friction is controlled largely by the trapping of ion atoms in the energy valleys near graphene atoms, friction is weaker at σ = −0.12 C/m^2^.

**Figure 5:**
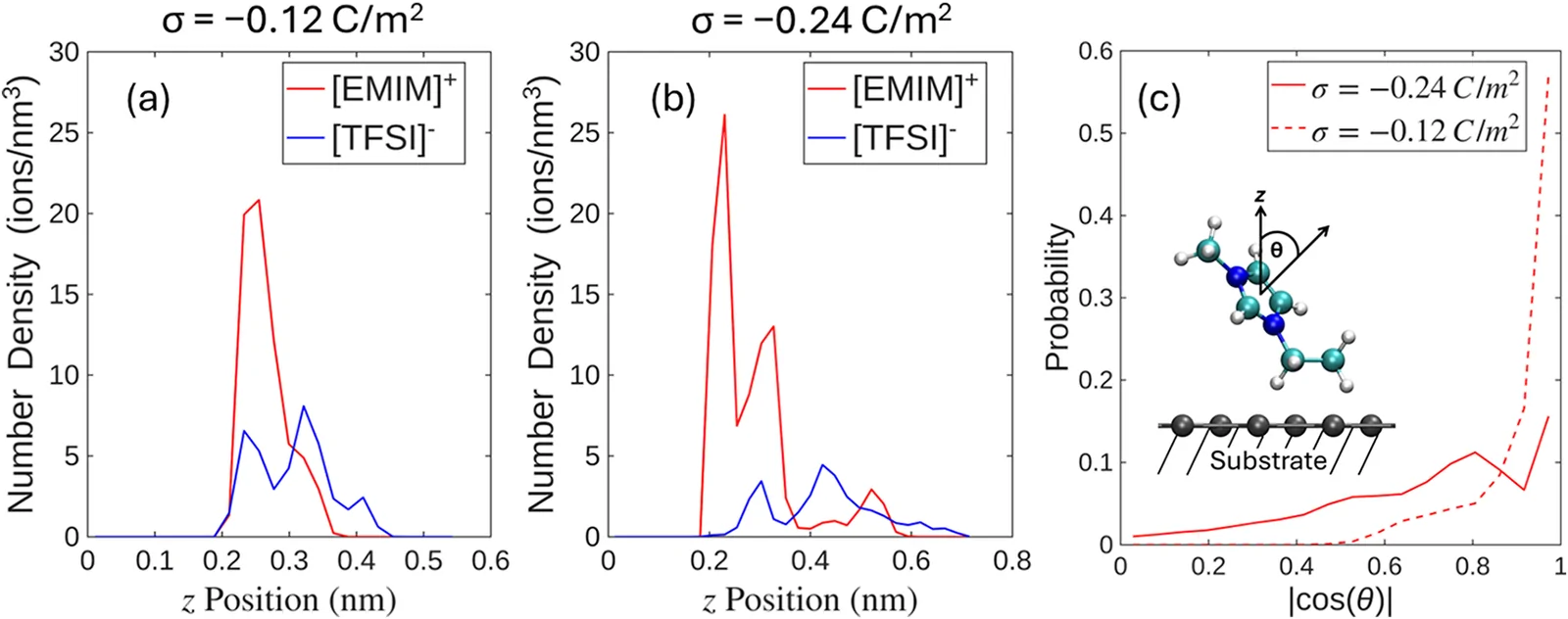
Number density profile of cations and anions across the nanogap formed by the graphene substrate and the tip apex for graphene surface charge densities of σ = −0.12 C/m^2^ (a) and σ = −0.24 C/m^2^ (b). (c) Comparison of the orientation of the imidazolium rings of cations trapped beneath the tip when the substrate charge is −0.12 and −0.24 C/m^2^.

When the substrate charge becomes more negative, i.e., σ = −0.24 C/m^2^, the number density of cation (anion) increases (decreases) further (see Figure 5b). The crowding of the space under the tip drives some cations to abandon their coplanar orientation relative to the substrate (see Figure 5c). This tilted orientation allows the cation atoms to penetrate deeper into the interstitial spaces between graphene atoms, leading to a significant contribution to friction. Consequently, as the surface charge decreases below σ = −0.12 C/m^2^, the dominant contribution to friction finally shifts from anions to cations. This shift is also corroborated by Figure 3b, which shows that, at σ = −0.24 C/m^2^, the number of cation atoms trapped in energy valleys near graphene is substantially higher than that of anion atoms.

The shift of the majority contributor to friction from anions to cations is further illustrated in Figure 6, which visualizes the spatial configuration of ions beneath the tip for σ ≤ 0 together with the number of atoms trapped in the substrate energy valley. The figure shows a clear increase in the population of trapped cation atoms as the surface charge becomes more negative, directly reflecting the shift in the major frictional contribution toward cations. Consequently, for surface charges more negative than σ = −0.12 C/m^2^, the combined increase in cation population and penetration depth, coupled with their stronger electrostatic attraction to the substrate, leads to a rapid rise in friction.

**Figure 6:**
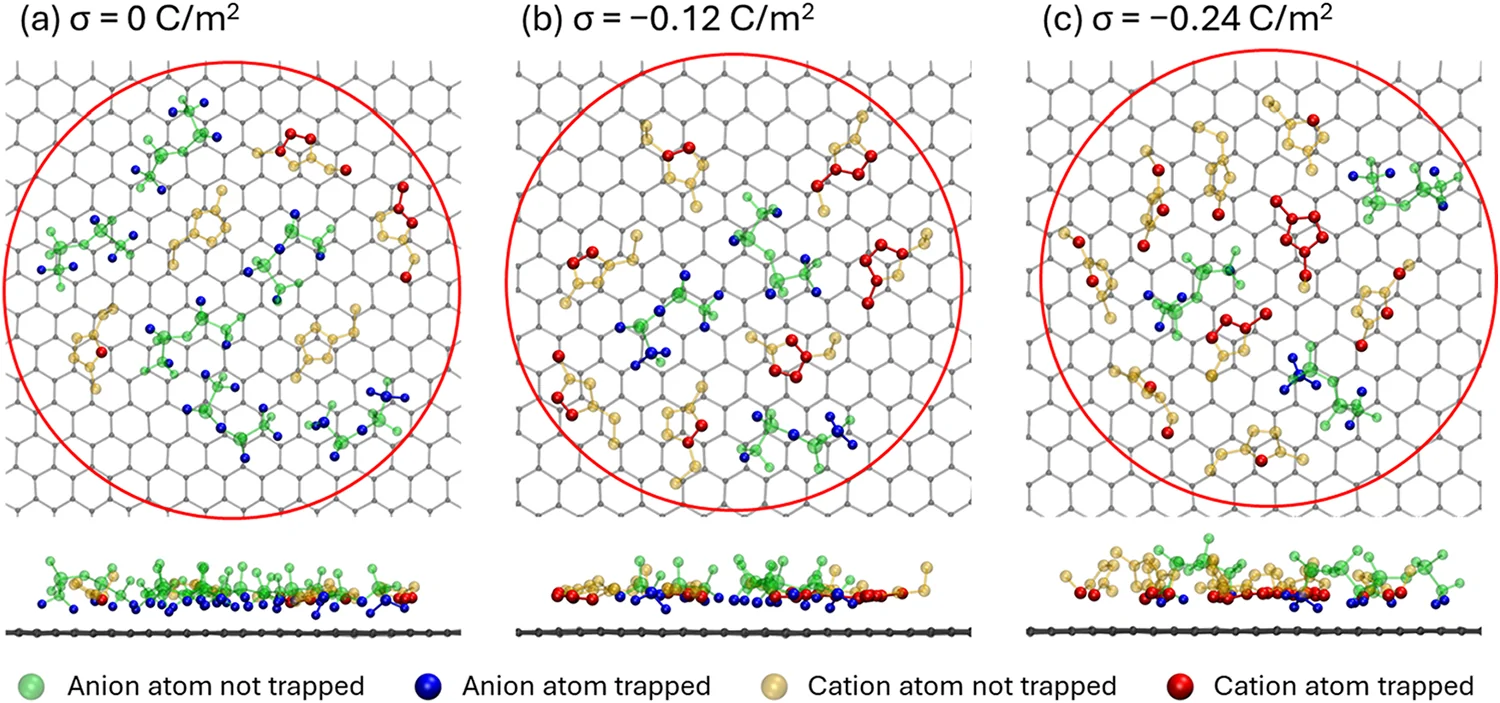
Representative top- and side-view snapshots of ions beneath the tip apex when the surface charge density of graphene is (a) σ = 0 C/m^2^, (b) σ = −0.12 C/m^2^, (c) σ = −0.24 C/m^2^. Ion atoms are color-coded based on whether they are trapped in the energy valleys between graphene atoms (see the text). For clarity, hydrogen atoms and ions not completely under the tip apex (marked by the red circle) are not shown.

The above variations of friction with graphene’s surface charge result in a friction minimum at a charge density of −0.12 C/m^2^ rather than zero, which is attributed largely to the size and shape asymmetries of the [EMIM]^+^ and [TFSI]^−^ ions. Conceivably, the deviation of the surface charge density at minimum friction from zero charge can also depend on the tip-ionic liquid interactions. This is because a variation in tip-ionic liquid interactions may change the confined ionic layer structure, alter friction, and thus shift the surface charge density associated with the friction minimum. The extent to which such a shift occurs warrants future studies.

### 3.3 Comparison of Friction Behaviors of [EMIM]­[TFSI] and [EMIM]­[TfO]

To ensure an appropriate comparison of the friction behaviors of [EMIM]­[TFSI] and [EMIM]­[TfO], the same normal load is applied to the tip. For both ionic liquids, a single ion layer under the tip is confirmed (see Figure S6), and the slip plane is found to be similar for both systems, i.e., at the ion–substrate interface. Figure 3a shows that the friction response of [EMIM]­[TfO] to graphene’s electrification follows the same trend as [EMIM]­[TFSI]: the friction force is lowest at a modest negative surface charge of σ = −0.12 C/m^2^, and on both sides, the friction force increases. Quantitatively, the left branch of the friction curve, i.e., from σ = −0.12 C/m^2^ to σ = −0.24 C/m^2^, is similar because these ionic liquids share the same cation. However, on the right branch, i.e., from σ = −0.12 C/m^2^ to σ = +0.24 C/m^2^, the friction is higher for [EMIM]­[TFSI]. This difference can be understood by examining the representative cases at a surface charge density of +0.24 C/m^2^.

We analyzed the last 40 ns of the trajectory and recorded the number of cations and anions in the gap under the tip at σ = +0.24 C/m^2^ for both systems. For [EMIM]­[TFSI], on average, 0.7 cations and 9 anions occupy the gap, whereas for [EMIM]­[TfO], the corresponding average numbers are 2 cations and 11 anions, respectively. The higher total ion population under the tip for [EMIM]­[TfO] than [EMIM]­[TFSI] is attributed to [TfO]^−^ ions’ smaller size (the volume of a [TfO]^−^ ion (0.117 nm^3^) is 47% smaller than that of a [TFSI]^−^ ion, 0.221 nm^3^).^47^ Note that because [EMIM]^+^ ions are repelled strongly from the gap, even though more space is available when the counterions in the gap are switched from [TFSI]^−^ to [TfO]^−^ ions, the increase of [EMIM]^+^ and [TfO]^−^ ion population under the tip is quite limited.

The increased total ion population under the tip for lubricant [EMIM]­[TfO], however, does not necessarily mean a higher friction since the latter is more directly controlled by the trapping of ion atoms by the energy valleys near the graphene substrate. Therefore, we further examined the number of atoms trapped in the graphene substrate’s energy valleys. Figure 7 shows the histogram of the number of cation and anion atoms trapped in energy valleys near the graphene substrate (an atom is considered trapped if its *z*-position falls below a threshold determined in Section 3.1, see the Supporting Information). We observe that, on average, 2.03 cation atoms are trapped in the case of [EMIM]­[TFSI] compared to 12.90 cation atoms in the case of [EMIM]­[TfO], i.e., the contribution of cations to friction increases when [TfO]^−^ ions are the co-ions under the tip. On the other hand, on average, 48.0 anion atoms are trapped in the case of [EMIM]­[TFSI] compared to 39.3 anion atoms in the case of [EMIM]­[TfO] despite the reduced number of [TFSI]^−^ ions under the tip (this is reasonable because a [TFSI]^−^ ion has 15 atoms, compared to 8 atoms of the [TfO]^−^ ion), i.e., the contribution of anion to friction decreases when [TfO]^−^ ions are the co-ions under the tip. However, as mentioned earlier, at high positive surface charge densities, cation atoms cannot reach deep into energy valleys due to [EMIM]^+^ ions’ coplanar configuration relative to the graphene substrate. Therefore, at σ = +0.24 C/m^2^, the friction force is reduced when [EMIM]­[TFSI] is replaced by [EMIM]­[TfO].

**Figure 7:**
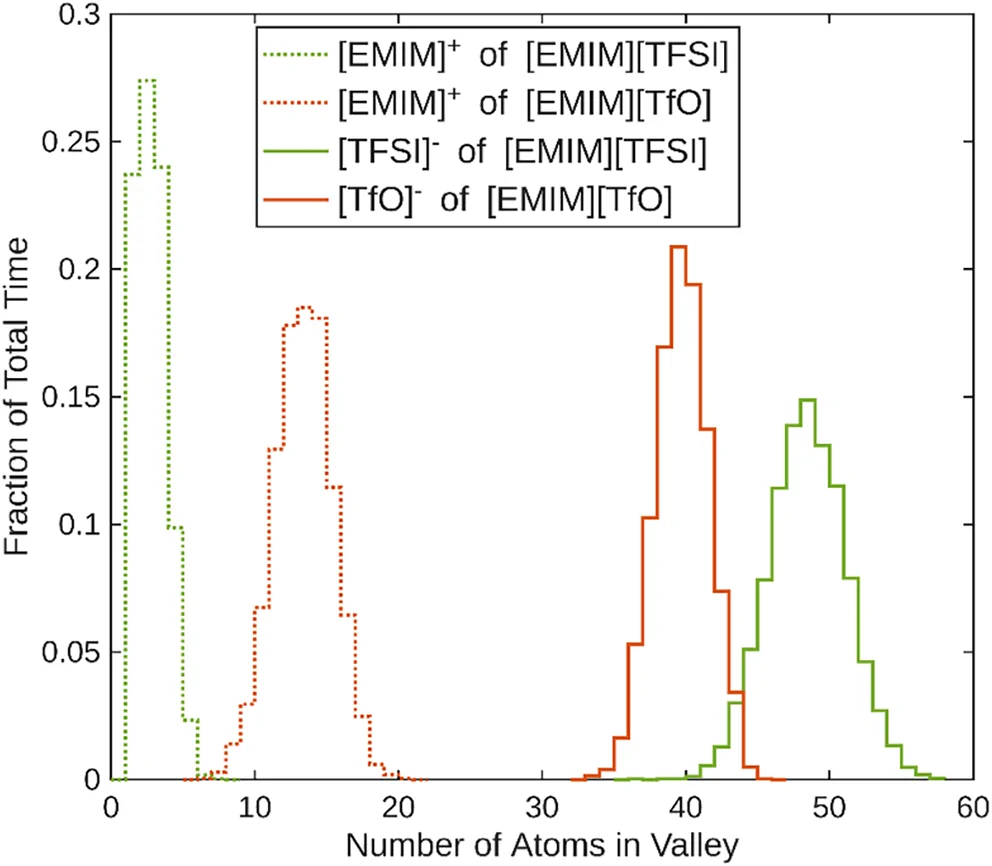
Histograms of the number of cation and anion atoms trapped in the energy valleys between graphene atoms at a surface charge density of +0.24 C/m^2^ when the ionic liquids are [EMIM]­[TFSI] and [EMIM]­[TfO].

## 4 Conclusions

In summary, the electrotunable friction is investigated within a system where a single layer of ionic liquids, [EMIM]­[TFSI] or [EMIM]­[TfO], is confined between a hemispherical tip and a flat graphene. Our all-atom molecular dynamics simulations show that friction vs surface charge density curves for both ionic liquids are V-shaped, with minimum friction observed at a surface charge density of −0.12 C/m^2^. When the surface charge density varies from −0.12 C/m^2^ to +0.24 C/m^2^, cations under the tip apex lie flat on the substrate and cannot penetrate the graphene substrate’s energy valley as deeply as anions. Consequently, more anion atoms are trapped in energy valleys in the interstitial space between graphene atoms, and anions dominate friction. The [TFSI]^−^ ions incur higher friction than the [TfO]^−^ ions due to their bulkier size and greater number of atoms. When the graphene surface charge density decreases from −0.12 C/m^2^ to −0.24 C/m^2^, anions are gradually depleted under the tip, and more cations under the tip apex tilt their ring relative to the graphene and penetrate deeper into graphene’s energy valleys. As such, the dominant contribution to friction shifts from anions to cations, and the friction for the two ionic liquids becomes similar.

Our all-atom simulations help understand the electrotunability of friction modulated by a single ion layer. The V-shaped friction vs surface charge density curves are in good agreement with those discovered through coarse-grained MD simulations^18^ involving [BMIM]­[PF_6_], where the [BMIM]^+^ ([PF_6_]^−^) ion was modeled as three (one) spherical beads. The fact that coarse-grained MD simulations capture key features of the electrotunability of friction robustly is encouraging. Indeed, because coarse-grained simulations are generally one to 2 orders of magnitude faster than their all-atom counterparts, their robust performance suggests that they may be used for building large data sets of the lubrication performance of ionic liquids. Such large data sets are indispensable in machine learning-accelerated discovery of lubricants, but have been difficult to obtain computationally due to the high computational cost of all-atom simulations.

## Supplementary Material



## Notes

Additional stick–slip analysis, *z*-position profiles of atoms below the AFM tip, stick–slip and instantaneous friction force in low sliding velocity, slip-plane analysis for [EMIM]­[TFSI], and number density plots for [EMIM]­[TfO]. (PDF)
