# Supplementary material for: All-Atom Molecular Dynamics Simulations of Electrotunability in Ionic Liquid Lubrication: Mechanism and Anion Specificity
Source: J Phys Chem C Nanomater Interfaces. 2026 Aug 13;130(34):12187–96. doi: 10.1021/acs.jpcc.6c03564 (PMC13528289; doi:10.1021/acs.jpcc.6c03564)
Supplement: Supplementary file 1 [file jp6c03564_si_001.pdf]

Supporting Information

All-Atom Molecular Dynamics Simulations of Electrotunability in Ionic Liquid Lubrication: Mechanism and Anion Specificity

Md Ashiqur Rahman[1], Wei Song[2], Ejaz Ahmed[1], Huajie Zhang[1], Akam Aboubakri[1], Rosa M. Espinosa-Marzal[2,3], and Rui Qiao[1,*]

[1] Department of Mechanical Engineering, Virginia Tech, Blacksburg, VA 24061, United States

[2] Department of Civil and Environmental Engineering, University of Illinois at Urbana-Champaign, Urbana 61801, United States

[3] Department of Materials Science and Engineering, University of Illinois at Urbana-Champaign, Urbana, Illinois 61801, United States

To find out the pattern of stick-slip of a trapped ion's atoms, the relative *x*-distance of the atoms of a specific cation and anion in the valley is tracked and shown in Figure S1. For the cation, hydrogen atoms are excluded from this analysis due to their rapid random vibration, which may provide misleading information. It is observed that for both cation and anion, the stick-slip cycle is a collective event. During the stick period, all constituent atoms remain stuck simultaneously. Subsequently, slip events are characterized by the simultaneous slippage of all atoms.

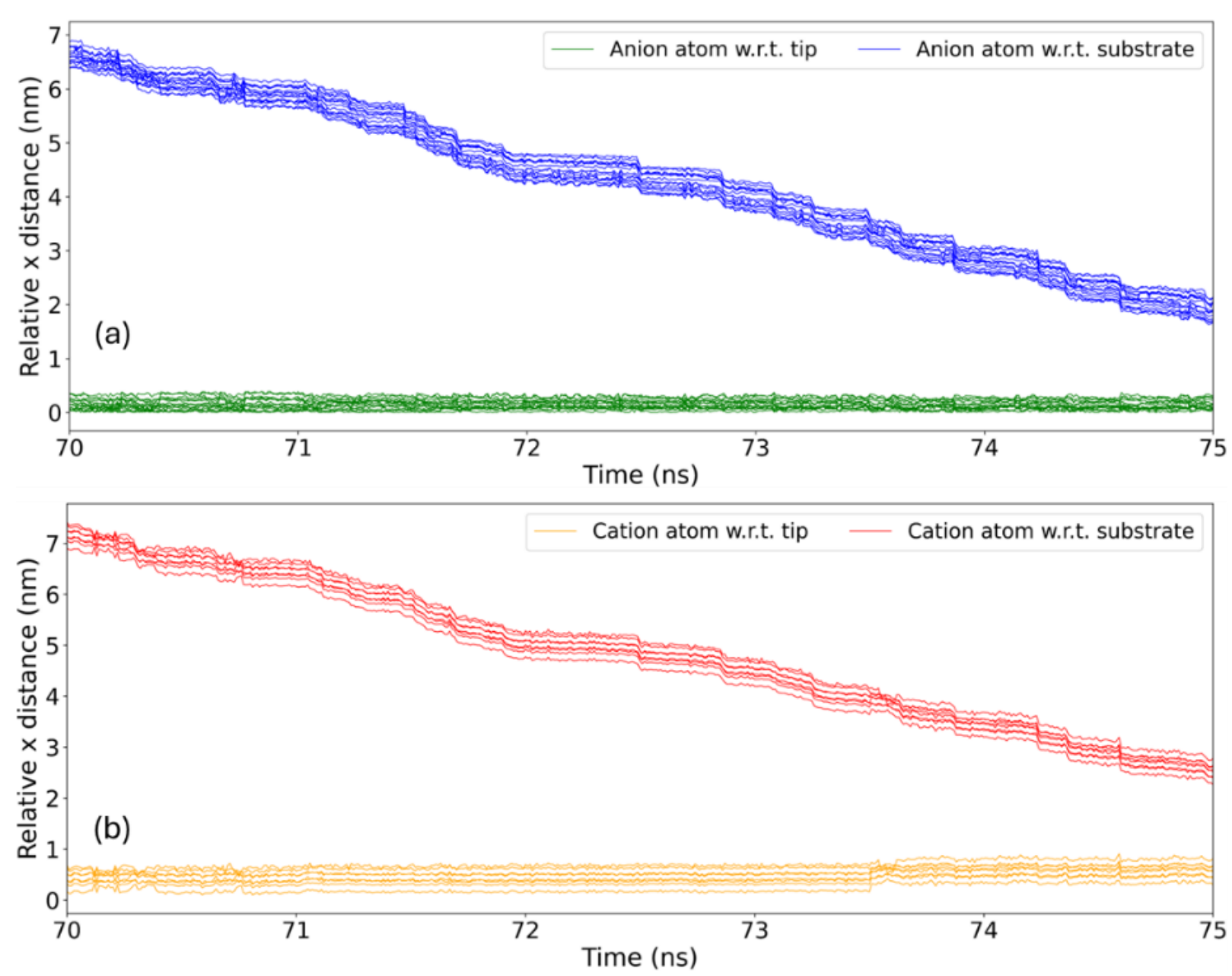

Figure S1. Relative *x*-distance of atoms of a particular trapped (a) anion, (b) cation with respect to the tip center and a fixed reference atom of the substrate.

* To whom correspondence should be addressed. Email: ruiqiao@vt.edu

A $[TFSI]^-$ ion beneath the tip apex is selected at t = 70 ns, and the relative *z*-positions of the four atoms in one of its CF_3 groups are monitored over 5 ns, as shown in Figure S2. Figure 2e of the main text presents the same results in a compact format, whereas the *z*-positions of the individual atoms are plotted separately here for clarity.

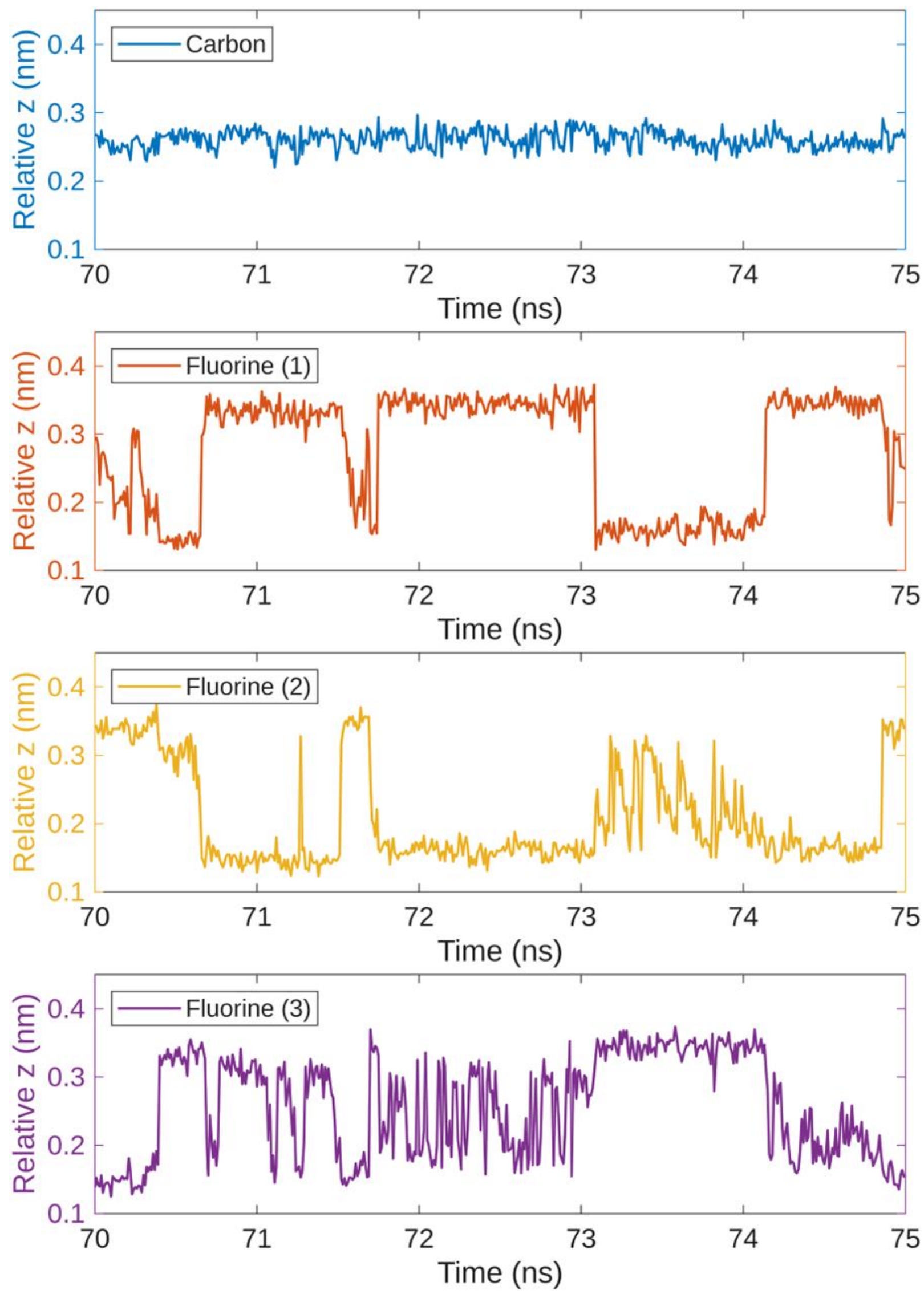

Figure S2. Relative *z*-position of atoms within a CF_3 group, measured from the upper substrate

The movement of the tip and the corresponding instantaneous friction force are examined at a pulling velocity of 0.1 nm/ns to investigate the stick-slip motion of the tip and its relationship with the friction force. Figure S3 shows that the stick-slip motion of the tip is generally accompanied by sudden changes in the friction force, consistent with the behavior observed in Figure 2f.

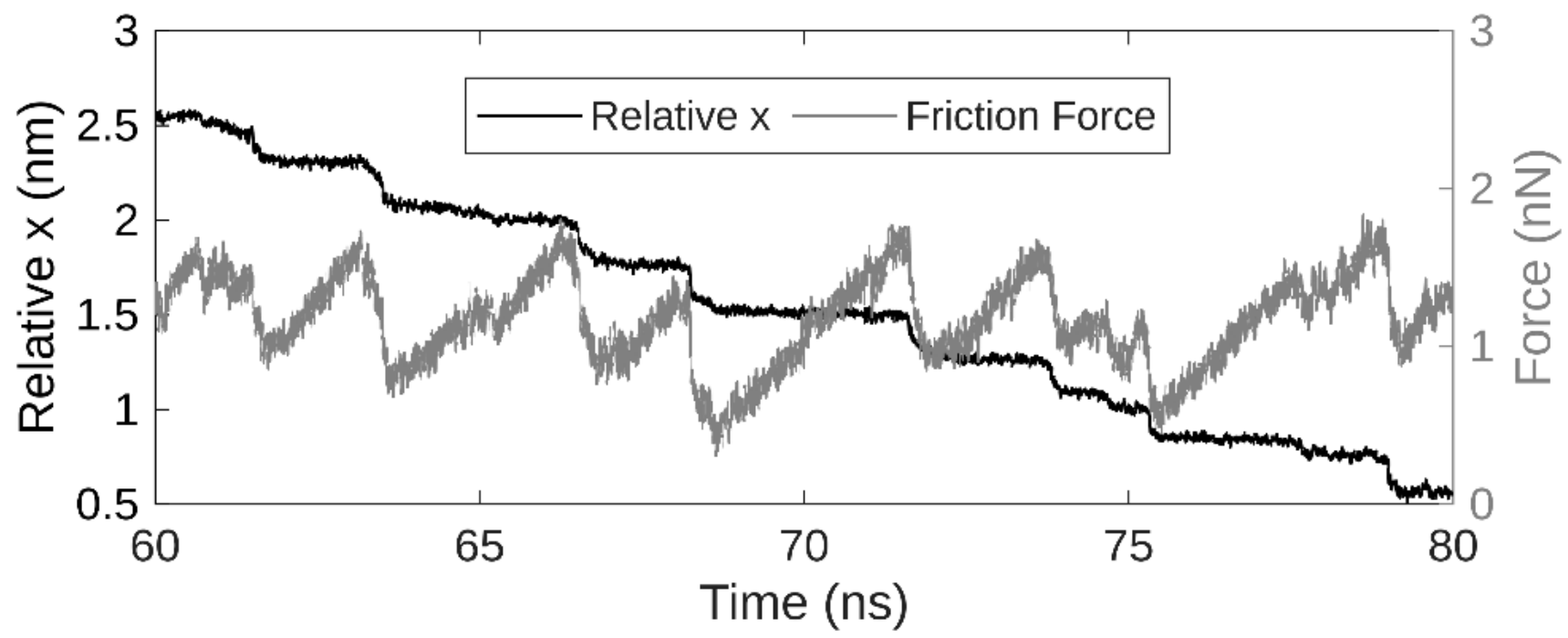

Figure S3. Relative x-position of the tip with respect to a fixed atom in the substrate and the corresponding instantaneous friction force with respect to time at a sliding velocity of 0.1 nm/ns.

To identify the threshold below which the atom can be considered to be trapped in the energy valley of graphene substrate, the relative z-position (from upper substrate) of the atoms under the tip is recorded for 40 ns while the tip is being pulled. Figure S4 illustrates the z-position of nitrogen, oxygen and fluorine atoms of the $[TFSI]^-$ anion. These atoms are observed to remain trapped within the energy valley, followed by a sudden jump when they escape from the valley. By correlating these events with trajectory visualization, the trapping thresholds for nitrogen, oxygen, and fluorine atoms are determined to be 0.24 nm, 0.22 nm, and 0.22 nm, respectively. A similar analysis is performed for all atom types to determine their corresponding trapping thresholds.

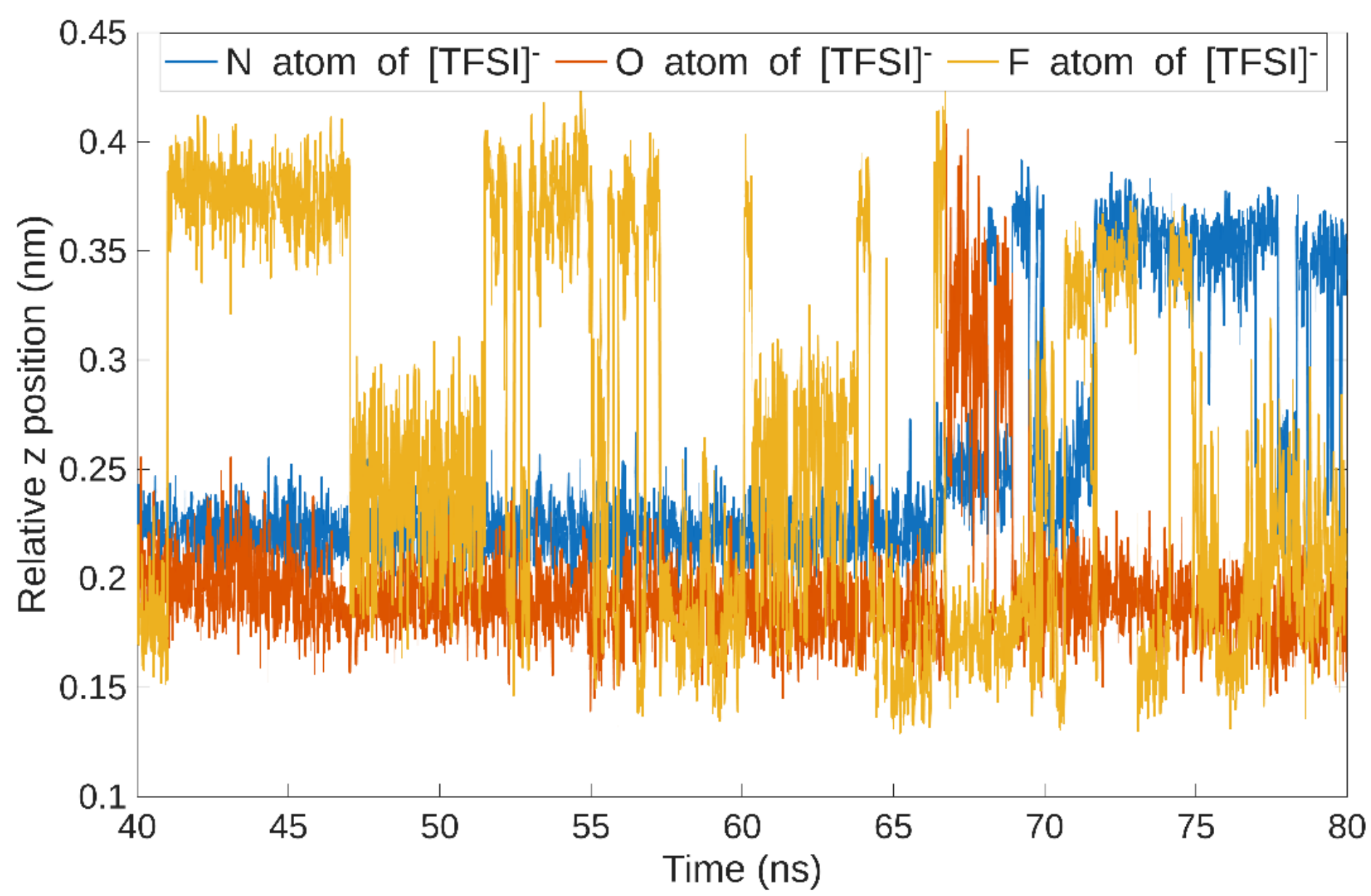

Figure S4. Relative *z*-position (from upper substrate) of nitrogen, oxygen and fluorine atoms of $[TFSI]^-$ anion atoms under the tip while the tip is being pulled.

Relative *x*-distance of ions beneath the tip apex in [EMIM][TFSI], measured with respect to the tip and the upper substrate layer, is analyzed and presented in Figure S5 to identify the slip plane when the tip is being pulled. In all cases, the relative distance is almost constant relative to the tip and changes relative to the substrate, which signifies that in all cases, the slip plane is between the IL and the substrate.

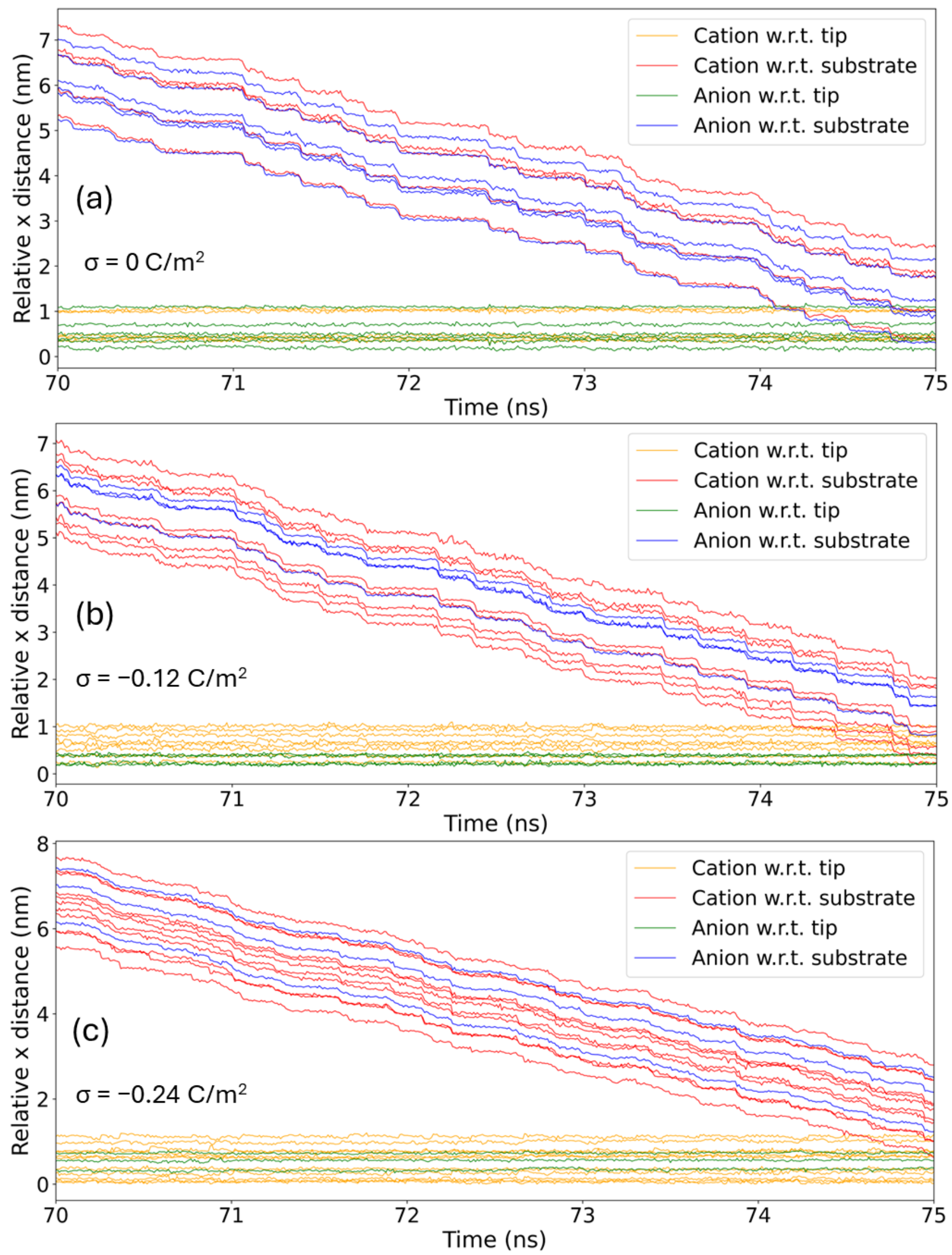

Figure S5. Relative x-distance of ions beneath the tip apex with respect to tip center and a fixed reference atom of the substrate when (a) $\sigma = 0$ C/m^2, (b) $\sigma = -0.12$ C/m^2, (c) $\sigma = -0.24$ C/m^2.

Number density of ions below the tip apex considering [EMIM][TfO] is analyzed for different surface charge density to ensure the presence of a single layer of IL. The graphs are presented in Figure S6.

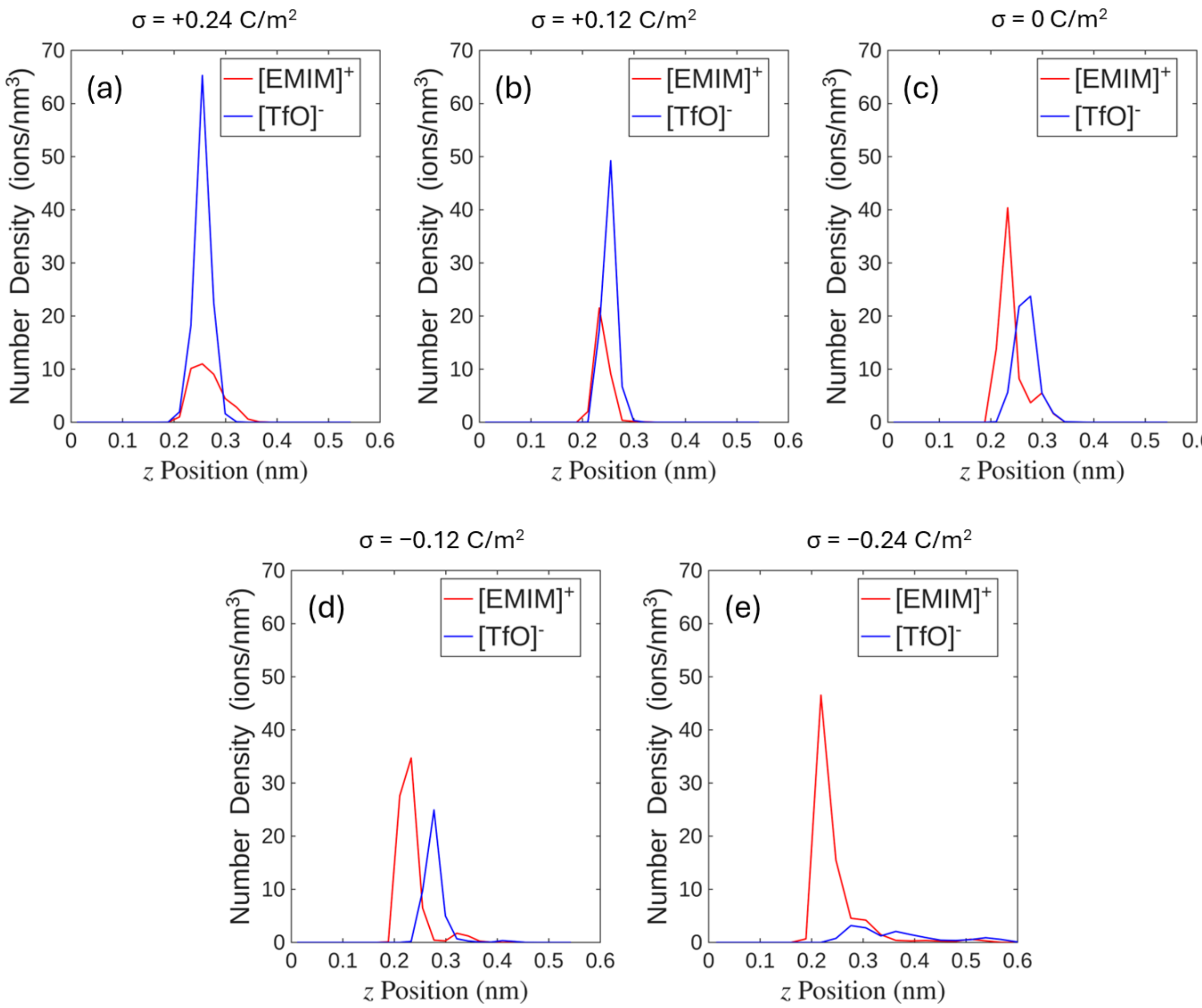

Figure S6. Number density of cation and anion for [EMIM][TfO] when (a) $\sigma = +0.24$ C/m^2, (b) $\sigma = +0.12$ C/m^2, (c) $\sigma = 0$ C/m^2, (d) $\sigma = -0.12$ C/m^2, (e) $\sigma = -0.24$ C/m^2. The cation is located by its ring center, and the anion is located by its sulphur atom.
